# Targeting Alpha‐Ketoglutarate Disruption Overcomes Immunoevasion and Improves PD‐1 Blockade Immunotherapy in Renal Cell Carcinoma

**DOI:** 10.1002/advs.202301975

**Published:** 2023-08-01

**Authors:** Le Li, Xing Zeng, Zheng Chao, Jing Luo, Wei Guan, Qiang Zhang, Yue Ge, Yanan Wang, Zezhong Xiong, Sheng Ma, Qiang Zhou, Junbiao Zhang, Jihua Tian, David Horne, Bertram Yuh, Zhiquan Hu, Gong‐Hong Wei, Baojun Wang, Xu Zhang, Peixiang Lan, Zhihua Wang

**Affiliations:** ^1^ Department of Urology Tongji Hospital Tongji Medical College Huazhong University of Science and Technology Wuhan 430030 China; ^2^ Institute of Organ Transplantation Tongji Hospital Tongji Medical College Huazhong University of Science and Technology Key Laboratory of Organ Transplantation Ministry of Education NHC Key Laboratory of Organ Transplantation Key Laboratory of Organ Transplantation Chinese Academy of Medical Sciences Wuhan 430030 China; ^3^ Institute of Reproductive Health Center for Reproductive Medicine Tongji Medical College Huazhong University of Science and Technology Wuhan 430030 P.R. China; ^4^ Department of Medicine Division of Hematology/Oncology Northwestern University Feinberg School of Medicine Chicago IL 60611 USA; ^5^ Department of Molecular Medicine Beckman Research Institute of City of Hope Duarte CA 91010 USA; ^6^ Fudan University Shanghai Cancer Center & MOE Key Laboratory of Metabolism and Molecular Medicine and Department of Biochemistry and Molecular Biology of School of Basic Medical Sciences Shanghai Medical College of Fudan University Shanghai 200032 China; ^7^ Department of Urology the Third Medical Center Chinese PLA General Hospital No.39 Yongding Road Beijing 100039 China

**Keywords:** alpha‐ketoglutarate, B2M, immunoevasion, PD‐1 blockade, renal cell carcinoma

## Abstract

The Warburg effect‐related metabolic dysfunction of the tricarboxylic acid (TCA) cycle has emerged as a hallmark of various solid tumors, particularly renal cell carcinoma (RCC). RCC is characterized by high immune infiltration and thus recommended for immunotherapeutic interventions at an advanced stage in clinical guidelines. Nevertheless, limited benefits of immunotherapy have prompted investigations into underlying mechanisms, leading to the proposal of metabolic dysregulation‐induced immunoevasion as a crucial contributor. In this study, a significant decrease is found in the abundance of alpha‐ketoglutarate (αKG), a crucial intermediate metabolite in the TCA cycle, which is correlated with higher grades and a worse prognosis in clinical RCC samples. Elevated levels of αKG promote major histocompatibility complex‐I (MHC‐I) antigen processing and presentation, as well as the expression of β2‐microglobulin (B2M). While αKG modulates broad‐spectrum demethylation activities of histone, the transcriptional upregulation of B2M is dependent on the demethylation of H3K4me1 in its promoter region. Furthermore, the combination of αKG supplementation and PD‐1 blockade leads to improved therapeutic efficacy and prolongs survival in murine models when compared to monotherapy. Overall, the findings elucidate the mechanisms of immune evasion in anti‐tumor immunotherapies and suggest a potential combinatorial treatment strategy in RCC.

## Graphical abstract

AKG disruption causes immunoevasion and αKG supplementation inhibits tumor growth in RCC. Mechanistically, αKG treatment decreases H3K4me1 in the promoter regions of B2M, thus upregulating MHC‐I expression and enhancing CTLs‐mediated anti‐tumor effects in multiple types of cancer including RCC. Combination treatment of αKG and PD‐1 blockade dramatically prolongs survival in mice, suggesting a novel promising combined immunotherapy approach.

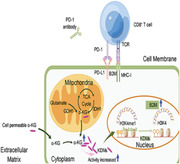

## 1 Introduction

Renal cell carcinoma (RCC) has long been characterized by dysregulated metabolic pathways and a highly infiltrated tumor immune microenvironment.^[^ ^1^ ^]^ Despite breakthroughs in surgical methods and targeted therapies including vascular endothelial growth factor inhibitors and rapamycin inhibitors or in combination with immune checkpoints blockade, oncologic outcomes still fall far short of expectations partly resulting from the high frequency of immunoevasion in RCC.^[^ ^2^ ^]^

Cancer cells frequently rewrite metabolic pathways to cope with the high metabolic demands of the tumor microenvironment (TME).^[^ ^3^ ^]^ Alterations to the tricarboxylic acid (TCA) cycle, an essential metabolic pathway for generating energy and biosynthetic intermediates, play a pivotal role in physiological and pathological states, including oncogenesis and inflammation.^[^ ^4^ ^]^ AKG, a key intermediate in the TCA cycle generated by oxidative decarboxylation of isocitrate with isocitrate dehydrogenase 1/2 (IDH1/2) enzyme or disproportionately replenished by deamination of glutamate with glutamate dehydrogenase 1 (GDH1) enzyme, has been reported to play multiple roles in various metabolic and cellular pathways.^[^ ^5^ ^]^

Recently, increasing attention has focused on whole genome‐wide epigenetic regulation and the corresponding physiological or pathological effects dominated by αKG and its derivatives.^[^ ^6^ ^]^ AKG is required by αKG‐dependent dioxygenases including **J**umonji C‐domain lysine demethylases (JmjC‐KDMs), ten‐eleven translocation (TET) DNA cytosine‐oxidizing enzymes, and prolyl hydroxylases (PHDs).^[^ ^7^ ^]^ Dysregulation of αKG‐dependent dioxygenases caused by mutations, amplifications, deletions, or silencing of their encoding genes, as well as hypoxia‐mediated indirect dysregulation, has been found to promote oncogenesis and tumor progression.^[^ ^6^ ^]^ Apart from direct regulation of tumor cell fate, αKG also manipulated metabolic and epigenetic reprogramming of immune cells in the TME and subsequently exerted impacts on cancer.^[^ ^8^ ^]^ Adoptive transfer of αKG‐treated Tregs into tumor‐bearing mice greatly enhanced immune infiltration and delayed tumor growth by altering mitochondrial metabolism and reshaping lipidome homeostasis in Foxp3^+^ Tregs.^[^ ^9^ ^]^ Moreover, mIDH1‐mediated inhibition of αKG‐dependent enzymes promoted immunoevasion and tumor maintenance in cholangiocarcinoma by decreasing CD8^+^ T‐cell recruitment and interferon γ (IFN‐γ) expression through the IFN‐γ‐TET2 axis.^[^ ^10^ ^]^ However, definitive evidence of αKG variations in malignant and benign tissues and the consequential effects on anti‐tumor immunity remain to be better defined in solid tumors.

In this study, we investigated the biological roles and immunomodulatory mechanisms of αKG in tumor cells with RCC as well as other malignant disease models to gain a more comprehensive view of connections between metabolites and immune regulation in hopes of developing novel effective combination therapeutic strategies.

## 2 Results

### 2.1 AKG is Decreased in RCC Tissues and Correlates with Malignancy in Humans and Mice

**Figure 1:**
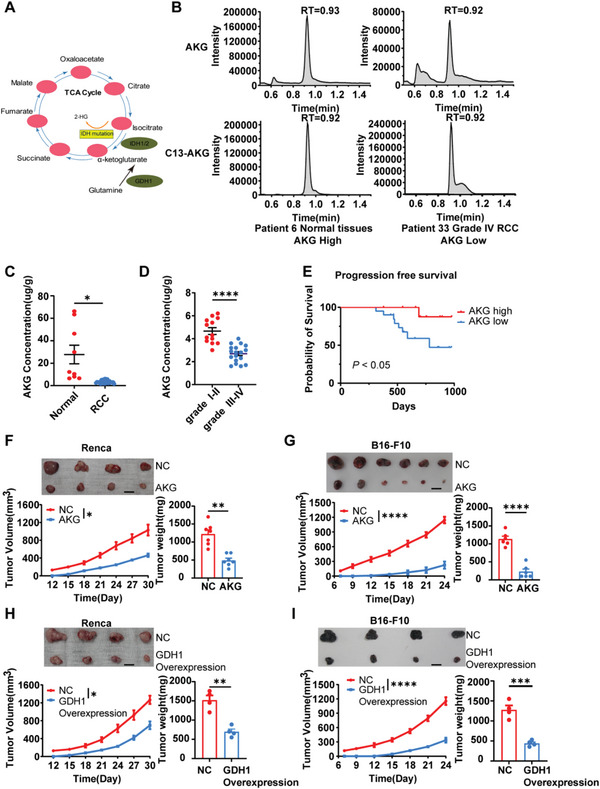
AKG attenuates Renal Cell Carcinoma development. A) The metabolic map of the α‐ketoglutarate metabolism pathway. B) Representative concentration of tumor αKG and C13‐αKG of two independent RCC patients. C) Quantification of αKG concentration in adjacent normal tissues (*n* = 9) and tumor tissues (*n* = 31) in RCC patients. Patients diagnosed with clear cell renal cell carcinoma were included (4 cm < mass diameter < 7 cm). D) Quantification of αKG concentration in tumor tissues of Grade I‐II (*n* = 13) and Grade III‐IV (*n* = 18) RCC patients. E) Progression‐free survival curves among patients with high concentration (no less than 4 µg g^−1^) and low concentration (< 4 µg g^−1^) αKG. *p* < 0.050. F) Average tumor growth and weight of Renca (*n* = 7) and G) B16‐F10 (*n* = 6) tumor‐bearing mice pre‐treated with/without 5 mm αKG for 4 days. H) Average tumor growth and weight of Renca (*n* = 4) and I) B16‐F10 (*n* = 4) tumor‐bearing mice transfected with vehicle (NC) or GDH1‐Overexpression plasmids. Data are mean ± S.E.M.; log‐rank (Mantel‐Cox) test was used for (E). Two‐tailed unpaired Student *t*‐test was used for (C,D) and (F–I). *p < 0.05, **p < 0.01, ***p < 0.001, and ****p < 0.0001. Scale bars represent 1 cm for (F–I).

AKG is a key intermediate in the TCA cycle (**Figure** **1**A). To investigate the putative roles of αKG and the effects of a disrupted TCA cycle in cancer, we examined the concentration of αKG in tissues derived from patients diagnosed with RCC. The levels of αKG were much lower in tumor specimens compared to adjacent normal samples (Figure 1B,C). Furthermore, intratumoral contents of αKG correlated with lower grades (Figure 1D) and better progression‐free survival rates (Figure 1E), implying a potential protective role of αKG in suppressing oncogenesis and tumor progression in RCC. It has been reported that GDH1 could produce αKG through its glutamate hydrogenation activity and that the knockdown of GDH1 could reduce intracellular levels of aKG.^[^ ^5^ ^]^ Consistent with this, we found that patients in the GDH1‐high group showed better prognosis and lower grades than those in the GDH1‐low group (Figure S1A,B, Supporting Information) using the TCGA‐KIRC dataset via Gepia program.^[^ ^11^ ^]^

To examine whether αKG is necessary to maintain the function of suppressing tumor development in mice models, RCC murine models subcutaneously transplanted with αKG‐treated and control tumor cells were established. Consistent with our clinical observations, αKG treatment and GDH1 overexpression delayed tumor growth in both murine RCC and melanoma models (Figure 1F‐I). Together, these results indicate that AKG attenuates RCC development.

### 2.2 AKG Promotes the Expression of B2M in RCC Tumors

**Figure 2:**
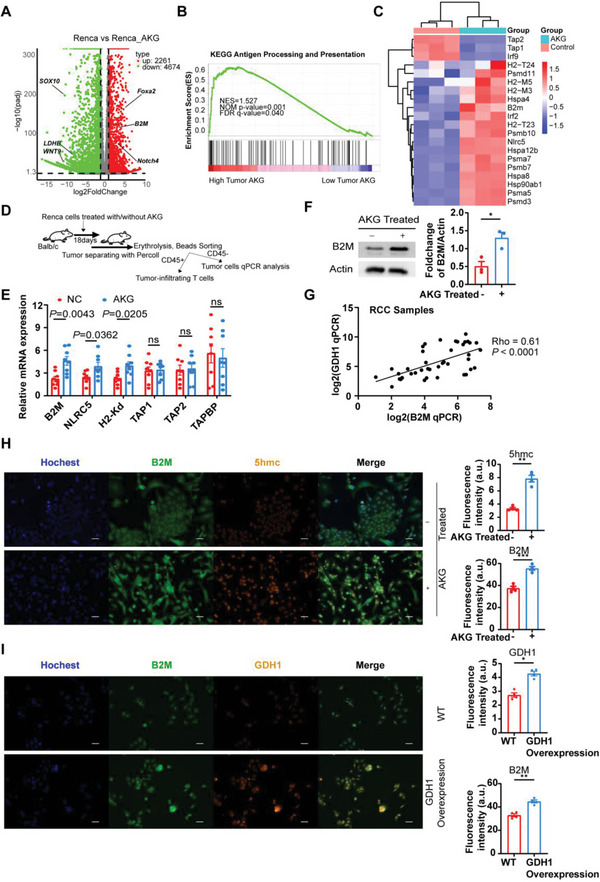
AKG promotes the expression of B2M in RCC tumors. A) Volcano plots showing DEGs of Renca cells (Fold change >1.5) between the αKG group and control group. B) GSEA enrichment results indicate the pathway enriched in the αKG high group. C) Heatmaps illustrate the expression of genes associated with antigen presentation and processing pathways between the αKG group and the control group. D) Schematic of experimental design for experiments shown in (E). E) qPCR analysis indicates the mRNA expression levels of common molecules (B2M, NLRC5, H2‐K^d^, TAP1, TAP2, TAPBP) associated with antigen presentation and processing pathways in Renca tumors between the αKG group and control group in vivo (*n* = 8). F) Representative and statistical western blot results (*n* = 3, in triplicate and repeated 3 times independently) show the protein levels of B2M of Renca cells between the αKG group and control group in vitro. G) Correlation of B2M and GDH1 mRNA expression in RCC clinical samples (*n* = 42). Statistical significance was determined by the Pearson correlation test. H) Representative and statistical analysis (*n* = 4, repeated 4 times independently) of the immunofluorescent staining results of nuclear (blue), B2M (green), 5hmc (red, localized in cellular nuclei), and merged files of Renca cells between the αKG group and control group. I) Representative and statistical analysis (*n* = 4, repeated 4 times independently) of the immunofluorescent staining results of nuclear (blue), B2M (green), GDH1 (red), and merged files of Renca cells between the GDH1‐Overexpression group and WT group. Error bar represents mean ± SEM. Statistical significance was determined by unpaired Student's *t*‐test for (E,F) and (H,I). ns = no significant, *p < 0.05, **p < 0.01, ***p < 0.001, and ****p < 0.0001. Scale bars represent 30 um for (H,I).

To decipher the mechanisms of suppressing roles of αKG in tumor development, we performed RNA‐sequencing on murine RCC Renca cells treated with or without cell‐permeable αKG, which showed up‐ and down‐regulated genes relevant to cell metabolism, cell fates, and cell components after αKG treatment (**Figure** **2**A). Additionally, gene set enrichment analysis (GSEA) showed enrichment of the “antigen processing and presentation pathway” in Renca cells treated with αKG (Figure 2B). Intriguingly, although the proteasome complex associated molecules (Psma5, Psma7, Psmb7, Psmd3) and key genes encoding basic components of MHC class I molecules such as B2M (comprising the light chain of MHC‐I), H2‐M3 and H2‐T23 were activated in Renca cells after αKG treatment,^[^ ^12^ ^]^ yet peptide transporters associated with antigen processing (TAP1, TAP2) did not follow this tendency (Figure 2C), suggesting the activated antigen processing and presentation pathway could be mainly attributed to elevated MHC‐I class molecules. Furthermore, we also analyzed the RNA‐seq data of cohort GSE167514 (human RCC cell lines 786O transfected with GDH1‐shRNA and Control‐shRNA) and GSE121580 (human glioma cell lines U87 transfected with GDH1‐shRNA and Control‐shRNA).^[^ ^13^ ^]^ Consistently, antigen presentation and processing pathways were enriched and B2M was elevated in the ShNT group with higher αKG levels (Figure S1C‐G, Supporting Information). To further investigate whether this transcript regulation would apply in vivo, we performed qPCR analysis on the tumor cells derived from RCC murine models (Figure 2D) and found B2M was also elevated upon αKG treatment (Figure 2E), suggesting that αKG‐induced upregulation of B2M were relatively steady within a certain period of days. Moreover, B2M overexpression was also elevated in vitro after αKG treatment (Figure 2F) and positively correlated with GDH1 expression in RCC clinical samples (Figure 2G) and KIRC database (Figure S1H, Supporting Information), accompanied by accumulation of 5hmc in the nucleus (Figure 2H) and GDH1 in the cytoplasm (Figure 2I), the molecules that are positively correlated with intracellular αKG contents.^[^ ^14^ ^]^ Taken together, these results strongly suggest that αKG can promote B2M expression in RCC Recal cells.

### 2.3 B2M is Lowly Expressed in RCC and Positively Correlates with Prognosis and CD8^+^ T Cell Infiltration

**Figure 3:**
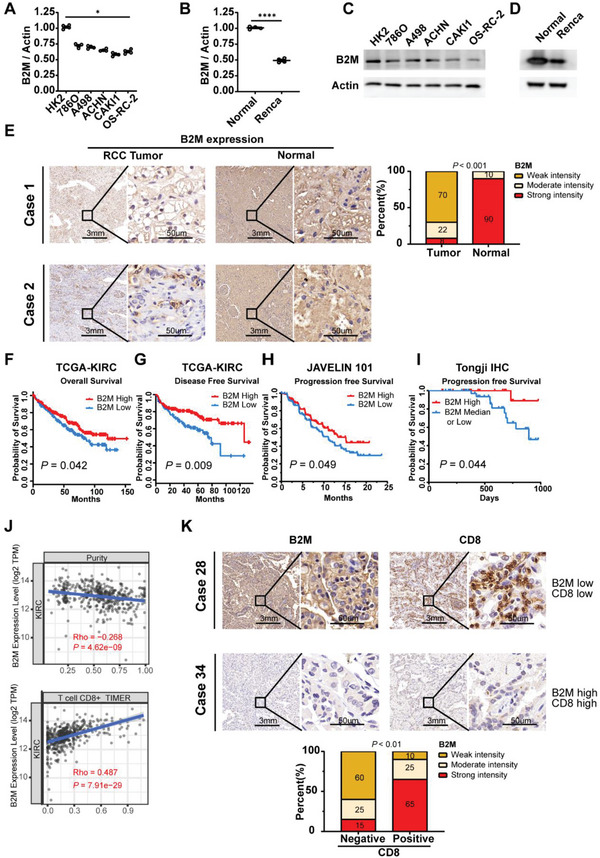
B2M is lowly expressed in Renal Cell Carcinoma and positively correlates with prognosis and PD‐L1 expression and CD8^+^ T cell infiltration. A) mRNA and C) protein expression of B2M between human renal cell carcinoma cell lines (786O, A498, ACHN, CAKI‐1 and OS‐RC‐2) and HK‐2 (human renal proximal convoluted tubule epithelial cell). B) mRNA and D) protein expression of Renca cell and normal kidney tissues from Balb/c mouse. E) Representative and statistical IHC results of B2M expression in RCC tumor (*n* = 70) samples and adjacent normal (*n* = 40) samples. F) Overall survival and G) Disease‐free survival of patients with high‐ or low‐B2M expression in the TCGA‐KIRC cohort using the Gepia 2.0 database (log‐rank test). H) Progression‐free survival of patients with high‐ or low‐B2M expression in the phase 3 JAVELIN Renal 101 trial (log‐rank test). I) Progression‐free survival of patients with high‐ or low‐B2M expression in our Tongji IHC samples. J) Correlation of B2M expression with tumor purity and infiltration levels of immune cells obtained from TIMER (purity‐corrected Spearman test). K) Representative and statistical IHC results of B2M (left) (*n* = 65), CD8 (right) (*n* = 55) in RCC tumors. Error bar represents mean ± SEM. Statistical significance was determined by unpaired Student's *t*‐test for A‐B and E and K. ns = no significant, *p < 0.05, **p < 0.01, ***p < 0.001, and ****p < 0.0001.

**Figure 4:**
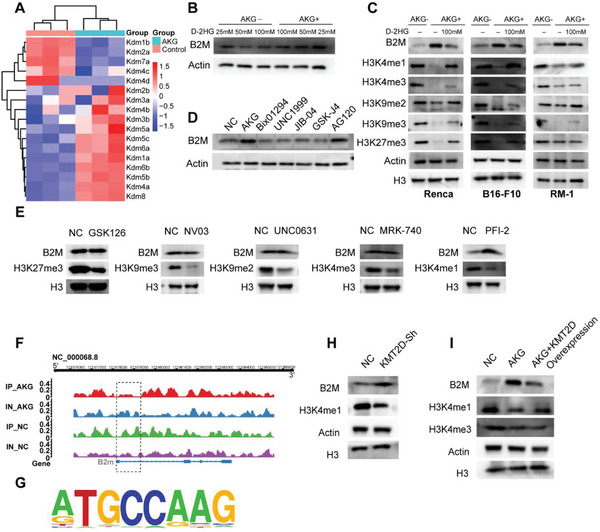
AKG upregulates B2M expression by debilitating enrichment of H3K4me1 in the promoter regions. A) Heatmaps illustrate the expression of genes in the αKG‐dependent histone demethylase family. B) Western results show B2M expression under the concentration of 25, 50, and 100 mm D‐2HG treatment treated with/without 5 mm αKG. C) Western results show B2M, H3K4me1, H3K4me3, H3K9me2, H3K9me3, and H3K27me3 in 3 different murine tumor cell lines: Renca, B16‐F10, and RM‐1 treated with/without 5 mm αKG and 100 mm D‐2HG. β‐actin and H3 were used as the reference protein. D)Western results show B2M expression in Renca cells treated with αKG, Bix01294, UNC1999, JIB‐04, GSK‐J4, and AG120. E) Western results show B2M and H3K4me1, H3K4me3, H3K9me2, H3K9me3, and H3K27me3 expression in Renca cells treated with GSK126, NV03, UNC0631, MRK‐740, and PFI‐2. F) Chip‐seq results show the H3K4me1 enrichment in the promoter region of B2M in Renca cells treated with/without αKG. IP‐AKG/NC: Chip‐seq pulled by H3K4me1 in the AKG/NC group; IN‐AKG/NC: Chip‐seq pulled by IgG in the AKG/NC group; G) Top consensus motif identified by HOMER with H3k4me1 peaks in (F). *p*‐Value = 1e‐39. H,I) B2M expression and H3K4me1 levels in Renca treated as indicated.

To further explore the role of B2M in RCC, we conducted qPCR and western blot analysis and found lower B2M expression in both murine Renca cells and human RCC tumor cells derived from distinct tissues of origin compared to normal kidney tissues/cells (**Figure** **3**A–D). Furthermore, human clinical paraffin‐embedded RCC tissues were also collected and immunohistochemical staining assays were performed, showing that the expression of B2M in tumor samples was much lower than that in cancer‐adjacent normal tissues (**Figure** **4**E). Using the Gepia database based on TCGA, we analyzed the correlation between B2M expression and the prognosis of KIRC and SKCM patients and found higher expression of B2M predicted better overall survival (OS) and disease‐free survival (DFS) (Figure 4F,G; Figure S2A, Supporting Information). Moreover, for the patients with advanced RCC treated with Avelumab (PD‐L1 antibody) from the phase 3 JAVELIN Renal 101 trial (n = 354; NCT02684006),^[^ ^15^ ^]^ higher expression of B2M also predicted better progression‐free survival (PFS) (Figure 4H). These results were in agreement with our IHC results based on patients from Tongji Hospital (Figure 4I). Low expression of B2M has long been considered as one of the recognized immune escape mechanisms in multiple tumors,^[^ ^16^ ^]^ however, the role of B2M in RCC still remains unclear. Using the public domain Timer 2.0 database,^[^ ^17^ ^]^ we found a positive correlation between B2M and infiltration levels of CD8^+^ T cells and DCs among multiple tumors including KIRC while no significant correlation was detected between B2M expression and tumor‐infiltrating CD4^+^ T cells (Figure 4J; Figure S2B‐D, Supporting Information). To verify this correlation, we used RCC IHC samples from Tongji Hospital and found that high expression of B2M was correlated with high infiltration levels of CD8^+^ T cells (Figure 4K), consistent with the results obtained from the Timer database.

Together, these results confirmed that B2M is lowly expressed in RCC and positively correlates with prognosis, possibly attributed to the increase of CD8^+^ T cell infiltration.

### 2.4 AKG Upregulates B2M by Attenuating the Enrichment of H3K4me1 in the Promoter Regions

Despite the characteristics of weak acids, pH values and acetate levels did not change significantly after αKG supplementation (Figure S3A,B, Supporting Information). As the key upstream enzyme of the TCA cycle, Pyruvate kinase M2 (PKM2) was overexpressed in RCC tumor samples (Figure S3C, Supporting Information), suggesting potential correlations of metabolic disruption with the whole TCA cycle.^[^ ^18^ ^]^ To further assess the influence of time and concentrations of αKG supplementation on B2M expression, we conducted western blot analysis and observed significantly elevated B2M expression in Renca cells treated with 5 mm αKG for 4 days (Figure S3D,E, Supporting Information). Additionally, RNA‐seq results showed that the majority of αKG‐dependent histone demethylases were increased in Renca cells upon treatment with αKG, including KDM1A (H3K4me1 demethylase), KDM3B (H3K9 demethylase), KDM4B (H3K9 and H3K36 demethylase), KDM5B (H3K4 demethylase) and KDM6A/6B (H3K27 demethylase) (Figure 4A).^[^ ^19^ ^]^ D‐2HG is a competitive inhibitor of αKG and inhibited the activity of αKG‐dependent histone demethylases in vitro.^[^ ^20^ ^]^ Supplementation of D‐2HG in cell‐culture medium decreased B2M expression in a dose‐dependent manner while adding both D‐2HG and αKG partly eliminated the inhibitory effects of D‐2HG on B2M expression (Figure 4B). Next, the three cancer cell lines: Renca‐RCC, B16‐F10‐Melanoma (both “hot tumors”) cells and RM1‐Prostate cancer cells (“cold tumor”) were used to confirm whether the αKG could induce histone demethylation and regulate the expression of B2M. Despite different extents of histone demethylation, B2M is overexpressed in all three murine cell lines (Figure 4C), suggesting that αKG regulates B2M expression in a wide variety of cancer rather than limited to RCC. Furthermore, supplementation of AG120, a small molecule inhibiting mutant IDH1 and lowering D‐2HG,^[^ ^21^ ^]^ exhibited a comparable capacity to upregulate the expression of B2M as well as αKG (Figure 4D). To investigate whether regulation of B2M expression by αKG is dependent on its histone demethylating capacity, we used several broad‐spectrum histone methylation and demethylation inhibitors: Bix01294 (decreases H3K9me2,3 and H3K36me2,3),^[^ ^22^ ^]^ UNC1999 (decreases H3K27me1,2,3 and H3K36me2),^[^ ^23^ ^]^ JIB‐04 (increases H3K4me1,2,3)^[^ ^24^ ^]^ and GSK‐J4 (increases H3K27me2,3 and H3K4me2,3)^[^ ^25^ ^]^ but did not observe changes of B2M expression (Figure 4D). Next, we used several selective histone methyltransferase inhibitors: GSK126 (decreases H3K27me3),^[^ ^26^ ^]^ NV03 (decreases H3K9me3),^[^ ^27^ ^]^ UNC0631(decreases H3K9me2),^[^ ^28^ ^]^ MRK‐740(decreases H3K4me3)^[^ ^29^ ^]^ and PFI‐2(decreases H3K4me1)^[^ ^30^ ^]^ and found that B2M was only overexpressed with concomitant hypomethylation of H3K4me1 (Figure 4E). To further elucidate the associations of H3K4me1 and B2M, we conducted CHIP‐seq analysis and observed a significant decrease of H3K4me1 in the promoter regions of B2M in Renca cells upon αKG treatment (Figure 4F,G). Additionally, knocking down H3K4 mono‐methyltransferase KMT2D^[^ ^31^ ^]^ with shRNA also promoted the upregulation of B2M (Figure 4H), which further demonstrated the epigenetic inhibition effects of H3K4me1 on B2M expression. Interestingly, increasing H3K4me1 using KDM5 inhibitor CPI455^[^ ^32^ ^]^ did not cause changes in B2M expression (Figure S3F, Supporting Information). Since histone deacetylases (HDACs) were also reported to regulate MHC‐I expression,^[^ ^33^ ^]^ we treated Renca cells with Nicotinamide (Class III HDACs) and trichostatin A (Class I/II/IV HDACs) and found trichostatin A rather than Nicotinamide (Figure S3G,H, Supporting Information) upregulated B2M expression, independent of the αKG‐mediated regulation on B2M (Figure S3I, Supporting Information). More importantly, overexpression of KMT2D prior to αKG treatment impaired the ability of αKG‐mediated demethylation of H3K4me1 and upregulation of B2M (Figure 4I), indicating αKG upregulates B2M by attenuating the enrichment of H3K4me1 in the promoter regions.

### 2.5 B2M Overexpression Inhibits Tumor Growth by Augmenting CD8^+^ T cell Infiltration and Cytotoxic Effects

**Figure 5:**
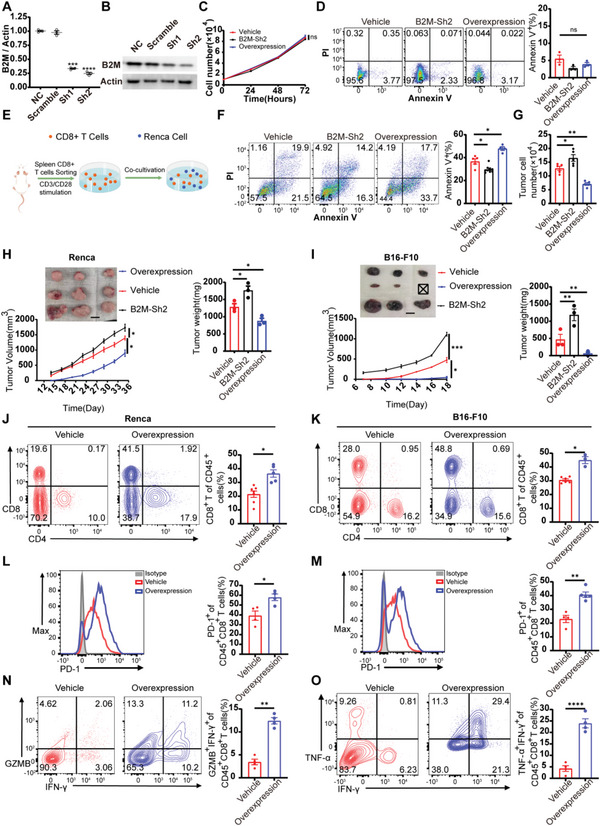
B2M overexpression inhibits tumor growth by augmenting CD8^+^ T cell infiltration and cytotoxic effects. A) qPCR (*n* = 3) and B) Western results show the shRNA knockdown efficiency for B2M. C) Renca cells growth assay using CCK8 to assess proliferation of Renca cells in the Vehicle group, B2M‐Sh2 group, and B2M‐Overexpression group (*n* = 4). D) Representative flow plots and quantification of Renca cells proportions stained with Annexin V and PI in the Vehicle group, B2M‐Sh2 group, and B2M‐Overexpression group (*n* = 3). E) A schematic representation of the co‐cultivation process. F) Representative flow plots and quantification of Renca cell proportions in different groups after co‐cultivation with CD8^+^ T cells (1:5) for 24 h. G) Remaining live cells (trypan blue‐negative) after co‐cultivation were counted by trypan blue assay and automated cell counting (*n* = 5). H) Renca cells and I) B16‐F10 cells were transfected with the vehicle, B2M‐Overexpression, and B2M‐Sh2 plasmids and transplanted subcutaneously to Balb/c and C57 mice (*n* = 3). Tumor volumes were measured every 2 or 3 days. On day 33 or 18, mice were sacrificed and tumor weight was analyzed. J,K) Representative flow plots and quantification of tumor‐infiltrating CD8^+^ T cells in Renca tumors of Vehicle group (*n* = 7) and B2M‐Overexpression group (*n* = 5) and B16 tumors of Vehicle group (*n* = 6) and B2M‐Overexpression group (*n* = 3). L,M) Representative flow plots and quantification of PD‐1 expression on the intratumoral CD8^+^ T cells of the Vehicle group (*n* = 4) and B2M‐Overexpression group (*n* = 4) from B16‐F10 tumor‐bearing mice and the Vehicle group (*n* = 4) and B2M‐Overexpression group (*n* = 5) from B16‐F10 tumor‐bearing mice. N,O) Representative flow plots and quantification of Granzyme B and IFN‐γ co‐expression and IFN‐γ and TNF‐α co‐expression in the intratumoral CD8^+^CD45^+^ T cells of the αKG group and control group from Renca and B16‐F10 tumor‐bearing mice (*n* = 4). Error bar represents mean ± SEM. Statistical significance was determined by unpaired Student's *t*‐test. ns = no significant, *p < 0.05, **p < 0.01, ***p < 0.001, and ****p < 0.0001. Scale bars represent 1 cm for (H,I).

To further explore the connections between B2M expression and CD8^+^ T cell‐mediated anti‐tumor effects, we constructed B2M‐Sh1 and B2M‐Sh2 plasmids and verified their knockdown efficiency using qPCR and western blots (**Figure** **5**A,B). After transfection with B2M‐Sh2, B2M‐Overexpression and vehicle plasmids, we conducted cell proliferation tests using the CCK8 kit and observed no significant differences (Figure 5C). Likewise, no significant apoptotic changes were observed after transfection with B2M‐Sh2, B2M‐Overexpression, and vehicle plasmids (Figure 5D). Next, we co‐cultivated Renca cells with CD8^+^ T cells by a 1:3 ratio for 24 hours (Figure 5E) and found that a higher Annexin V positive proportion in the Renca cells transfected with the B2M‐Overexpression group while the remaining tumor cell numbers were lower than those in the vehicle group (Figure 5F,G). Furthermore, the B2M‐Overexpression group exhibited smaller tumor volumes and lighter tumor weight than those in the vehicle and B2M‐Sh2 groups in both murine models of “hot tumor” (Figure 5H,I).^[^ ^34^ ^]^ Next, we analyzed the characteristics of tumor‐infiltrating lymphocytes (TILs) and found similar results: the infiltration proportions of CD8^+^ T cells rather than CD4^+^ T cells were significantly increased (Figure 5J,K) and the expression of PD‐1 in CD8^+^ T cells was also increased in the B2M‐Overexpression group compared to the vehicle group (Figure 5L,M). Moreover, dual positive cytokines (TNF‐α and IFN‐γ) and granzyme‐B producer CD8^+^ CD45^+^ T cell percentages were also increased (Figure 5N,O). Altogether, these results indicate that B2M overexpression inhibits tumor growth by augmenting CD8^+^ T cell infiltration and cytotoxic effects.

### 2.6 The αKG‐B2M Cascade Increases the Infiltration and Cytokine Production of Therapeutic CD8^+^ T Cells

**Figure 6:**
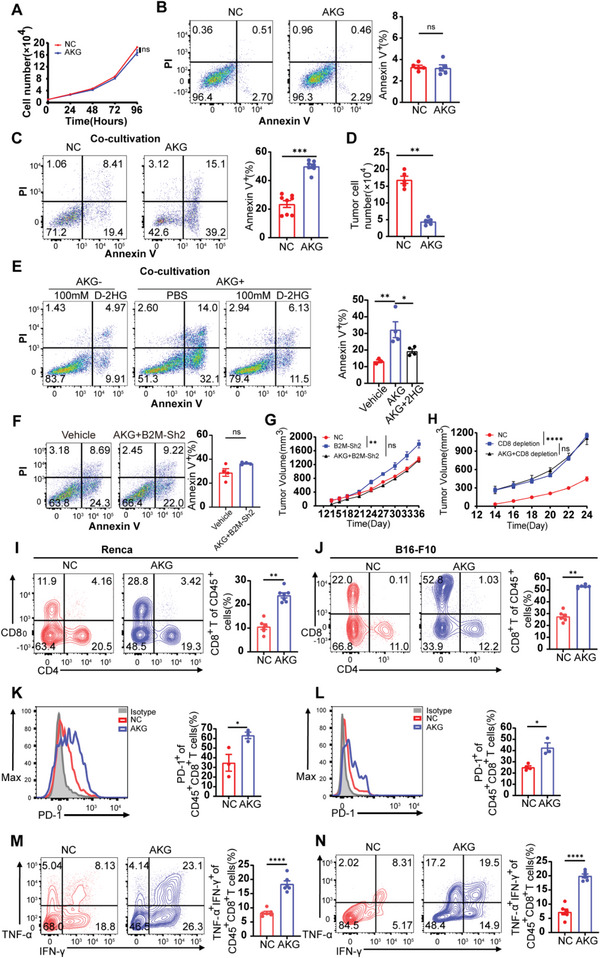
The tumor suppressor role of αKG is dependent on αKG ‐B2M axis enhanced anti‐tumor immunity. A) Renca cell growth assay using CCK8 to assess proliferation of Renca cells treated with/without αKG. B) Representative flow plots and quantification of Renca cells proportions stained with Annexin V and PI in the αKG group treated with 5 mm αKG and control group (*n* = 5). C) Representative flow plots and quantification of Renca cell proportions in the αKG group treated with 5 mm αKG and control group after co‐cultivation with CD8^+^ T cells (1:5) for 24 h. D) Remaining live cells (trypan blue‐negative) after co‐cultivation were counted by trypan blue assay and automated cell counting (*n* = 5). E) Representative flow plots and quantification of Renca cell proportions stained with Annexin V and PI in the D‐2HG group, αKG group, and αKG+D‐2HG group. F) Representative flow plots and quantification of Renca cell proportions stained with Annexin V and PI in the Vehicle group and αKG+B2M‐Sh2 group. G) Renca cells were treated with/without 5 mm αKG for 4 days and transfected with a vehicle, B2M‐Sh2 plasmids AND then transplanted subcutaneously to Balb/c mice (*n* = 3). Tumor volumes were measured every 2 or 3 days. H) Renca cells were treated with/without 5 mm αKG for 4 days and transplanted subcutaneously to Balb/c mice (*n* = 3). Mice were intraperitoneally injected with 100 µg of anti‐CD8 (the αKG group) or control antibody (Rat IgG) 1 day before and 4, 10 days after tumor implantation until harvest. Tumor volumes were measured every 2 or 3 days. I,J) Representative flow plots and quantification of tumor‐infiltrating CD8^+^ T cells in Renca tumors of αKG group (*n* = 7) and control group (*n* = 6) and B16 tumors of αKG group (*n* = 6) and control group (*n* = 6). K,L) Representative flow plots and quantification of PD‐1 expression on the intratumoral CD8^+^ T cells of the αKG group (*n* = 3) and control group (*n* = 3) from Renca tumor‐bearing mice and the αKG group (*n* = 4) and control group (*n* = 3) from B16‐F10 tumor‐bearing mice. M,N) Representative flow plots and quantification of IFN‐γ and TNF‐α co‐expression in the intra‐tumoral CD8^+^CD45^+^ T cells of the αKG group and control group from Renca and B16‐F10 tumor‐bearing mice (*n* = 6). Representative data are shown from two independent experiments. Error bar represents mean ± SEM. Statistical significance was determined by unpaired Student's *t*‐test. ns = no significant, *p < 0.05, **p < 0.01, ***p < 0.001, and ****p < 0.0001.

To explore the direct effects of αKG on tumor cells, we added cell‐permeable αKG to complete medium culturing Renca cells and found similar growth rates between αKG treated groups and controls (**Figure** **6**A). Analogously, 5 mm αKG neither induced apoptosis (early nor late) of Renca cells (Figure 6B). To further investigate whether the elevated αKG in tumor cells might cause differences in anti‐tumor immune responses, we collected activated CD8^+^ T cells and co‐cultured them with αKG‐treated Renca cells at a 2:1, 3:1, and 5:1 ratio for 24 h, respectively, and the results showed that CD8^+^ T cells exerted dramatically stronger cytotoxicity against αKG‐treated Renca cells compared to the control group at any co‐incubation rates (Figure 6C; Figure S4A, Supporting Information). Residual tumor cell numbers were counted from the co‐cultivation system and the Renca cells escaping from CTL killing were fewer in the αKG‐treated group (Figure 6D). To assess the alterations of CTL cytotoxicity towards tumor cells aroused from B2M expression change induced by D‐2HG and αKG, we co‐cultivated CD8^+^ T cells with Renca cells treated with αKG and D‐2HG alone or together. As expected, Renca cells underwent the mildest T cell killing in the D‐2HG group while the heaviest T cell killing occurred in the αKG group, and supplementation of both αKG and D‐2HG reduced the cytotoxic effects of CD8^+^ T cells compared to the αKG group (Figure 6E). Furthermore, the enhanced cytotoxic effects after αKG supplementation disappeared in the B2M‐Sh2 group (Figure 6F), further underlying the fact that the augmented cytotoxic effects were dependent on the upregulation of B2M. Next, we constructed murine models and found the αKG treatment could overcome immunoevasion caused by B2M knockdown (Figure 6G), but was unable to inhibit tumor growth after CD8 depletion in vivo (Figure 6H), suggesting αKG‐induced anti‐tumor effects rely on the downstream elevated expression of B2M and CD8^+^ T cell functions.

To gain a more comprehensive understanding of the alterations in antitumor immunity, we next sought to assess the profiles of major immune cell subgroups in the TME and observed higher numbers of tumor‐infiltrating CD8^+^ T cells in both murine tumor models (Figure 6I,J) but did not observe similar differences in the spleen (Figure S4B, Supporting Information). Conversely, proportions of other tumor‐infiltrating subgroups of immune cells like CD4^+^ T cells, NK cells, and tumor‐associated macrophages (TAMs) all exhibited no significant disparities while proportions of dendritic cells (DCs) were increased in the AKG group (Figure S4C‐G, Supporting Information). Moreover, we also found that the intra‐tumoral CD8^+^ T cells rather than CD4^+^ T cells in the αKG‐treated groups exhibited higher PD‐1 expression than those in the control groups (Figure 6K,L; Figure S4H, Supporting Information). We investigated the ability of CD8^+^ T cells to produce cytokine tumor necrosis factor‐α (TNF‐α) following terminal differentiation, which typically results in reduced TNF‐α secretion, but continued secretion of interferon‐γ (IFN‐γ). Through analysis of CD45^+^CD8^+^ T cells, we identified a higher proportion of TNF‐α^+^ IFN‐γ^+^ double‐positive cells in the groups treated with αKG. This supports the conclusion that αKG‐treated tumor cells can promote a killer phenotype in CD8^+^ TILs (Figure 6M,N). Altogether, these results demonstrate that the tumor suppressor role of αKG is dependent on the αKG‐B2M‐CD8^+^ T cell axis.

### 2.7 The Combination of αKG Treatment with PD‐1 Blockade Significantly Reduces Tumor Growth

**Figure 7:**
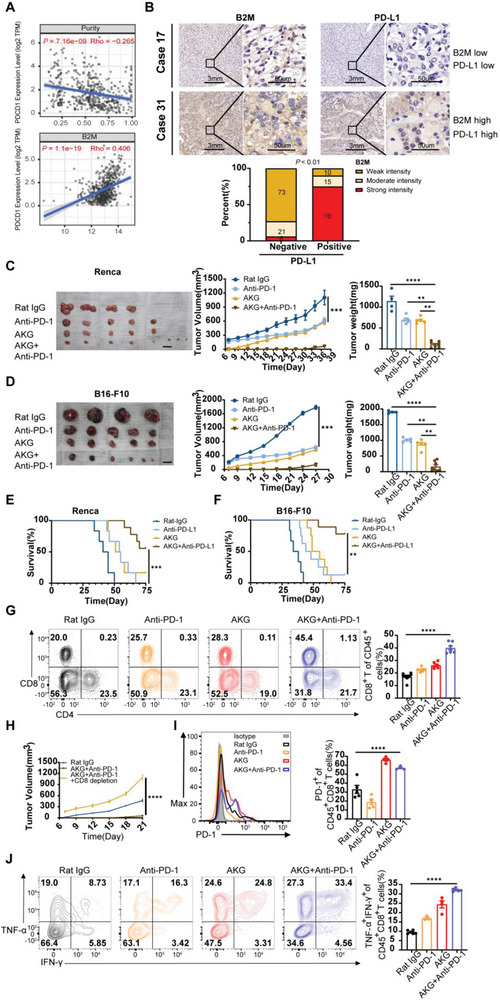
AKG potentiated anti‐PD1 immune checkpoint therapeutic efficacy. A) Correlation of B2M expression with tumor purity and PD‐1 expression of tumor‐infiltrating immune cells obtained from TIMER (purity‐corrected Spearman test). B) Top: IHC staining of B2M (left) and PD‐L1(right) in KIRC tumors collected by us was performed. Bottom: Quantitative IHC analysis of correlation of B2M expression (*n* = 65) and PD‐L1 expression (*n* = 50). C) Average tumor volumes and weight of Renca tumor‐bearing Balb/c mice and B16‐F10 tumor‐bearing C57 mice D) treated with Rat‐IgG (*n* = 4), αKG (*n* = 6), or anti‐PD‐1(*n* = 5) monotherapy and combination therapy (*n* = 8 in the Renca murine model and *n* = 6 in the B16‐F10 murine model). E) Survival curves of Renca tumor‐bearing Balb/c mice and F) B16‐F10 tumor‐bearing C57 mice treated as indicated. Renca model: Rat IgG group (*n* = 6), αKG group (*n* = 6), PD‐1 group (*n* = 6), and combination group (*n* = 9); B16‐F10 model: Rat IgG group (*n* = 6), αKG group (*n* = 8), PD‐1 group (*n* = 8), and combination group (*n* = 9). G) Representative flow plots and quantification of tumor‐infiltrating CD4^+^ and CD8^+^ T cells in Renca tumors and B16‐F10 tumors treated as indicated (*n* = 4). H) Average tumor growth of B16‐F10 tumor‐bearing animals treated with Rat IgG, combination therapy, and α‐CD8 depleting antibody. I) Representative flow plots and quantification of PD‐1 expression on the intra‐tumoral CD8^+^ T cells from B16‐F10 tumor‐bearing mice treated as indicated (*n* = 8 for Rat IgG group, *n* = 6 for αKG group, *n* = 6 for anti‐PD‐1 group, *n* = 7 for combination group). J) Representative flow plots and quantification of IFN‐γ and TNF‐α co‐expression in the intratumoral CD8^+^CD45^+^ T cells of the αKG group and control group from Renca and B16‐F10 tumor‐bearing mice (*n* = 5 for Rat IgG group, *n* = 3 for αKG group, *n* = 4 for anti‐PD‐1 group, *n* = 6 for combination group). Error bars represent S.E.M. log‐rank (Mantel–Cox) test was used for (E) and (F). Two‐way ANOVA was used for (C), (D), (G), (H), (I), and (J). *p < 0.05, **p < 0.01, ***p < 0.001, and ****p < 0.0001. Scale bars represent 1 cm for (C,D).

**Figure 8:**
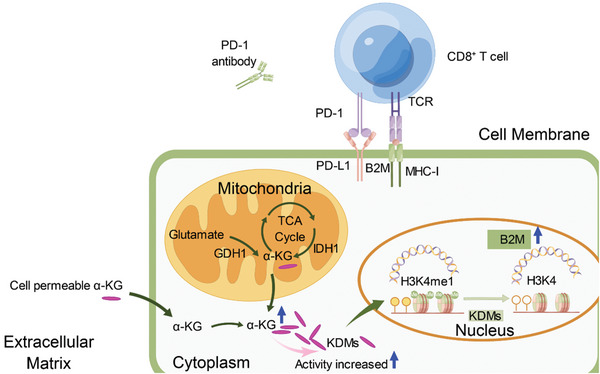
The αKG‐B2M‐CD8 axis creates therapeutic vulnerabilities in renal cell carcinoma. Schematic model depicting that enhancement of the αKG‐B2M‐CD8 axis improved PD‐1 blockade efficacy and inhibited tumor growth in renal cell carcinoma.

Although B2M overexpression augmented cytotoxic effects and promoted the infiltration of CD8^+^ T cells in our previous results, we also observed a concurrent upregulation of the PD‐1/PD‐L1 pathway in the public database Timer 2.0 (**Figure** **7**A), as well as in our clinical IHC results (Figure 7B) and previous murine models. To further investigate the potential associations between αKG treatment and PD‐1 blockade, we implanted mice subcutaneously with Renca cells and B16‐F10 cells treated with complete medium with or without αKG for 4 days. Following tumor initiation, mice were treated with anti‐PD‐1 antibody or rat IgG antibody as isotype controls. Therapy with anti‐PD‐1 antibody or αKG treatment alone both inhibited tumor growth in two respective murine models, while combination therapy of anti‐PD‐1 antibody and αKG treatment further delayed tumor growth or even led to tumor regression (Figure 7C,D) and significantly increased overall survival compared with monotherapy (Figure 7E,F). Furthermore, the proportions of intratumoral CD8^+^ T cells were significantly increased in the combination group (Figure 7G). To confirm the importance of CD8^+^ T cells in the combination group, we depleted CD8^+^ T cells after tumor transplantation and observed rapid re‐progression of the tumor (Figure 7H), indicating the critical role of CD8^+^ T cells in the significant anti‐tumor effects produced by combination treatment of αKG and anti‐PD1. Moreover, the percentages of PD1^+^ CD8^+^ T cells were comparable between the control group and combination treatment group (Figure 7I). Additionally, TNF‐α^+^IFN‐γ^+^CD8^+^CD45^+^ T cells were dramatically increased in the combination group (Figure 7J). Altogether, these results further demonstrate the tremendous potential of this combined modality to treat “hot tumors” including RCC and melanomas. This αKG‐B2M‐CD8 axis provides an explanation of the common immune evasion in anti‐tumor immunotherapies and creates therapeutic vulnerabilities in RCC (**Figure** **8**).

## 3 Discussion

The last decade has witnessed dramatic clinical success in terms of PD‐1 blockade therapy in multiple solid malignancies,^[^ ^35^ ^]^ yet only a proportion of patients benefit from this strategy while a substantial subset of patients either fail to respond or progress rapidly to resistance after initial response.^[^ ^36^ ^]^ Immunoevasion caused by the downregulation of MHC‐I class molecules has been proposed as a potential mechanism underlying this limited success.^[^ ^37^ ^]^ To improve the efficacy of ICB in solid tumors, investigations have focused on developing effective, feasible, low‐cost, and non‐toxic approaches to upregulating the expression levels of MHC‐I molecules and thus inhibiting tumor growth.^[^ ^37^ ^]^

We demonstrated that manual increase of intracellular αKG could be a stable and feasible way to upregulate B2M and MHC‐I of tumors through histone demethylation. AKG was already known to install a hypomethylation state to histones and DNA and then regulate the transcription of downstream genes.^[^ ^38^ ^]^ The extent and specific sites of the αKG‐dependent Jumonji‐C histone demethylating activities vary among different cells and tissues.^[^ ^38^ ^]^ In bone marrow mesenchymal stromal/stem cells (MSCs), αKG upregulated BMP signaling and Nanog expression by decreasing the accumulations of H3K9me3 and H3K27me3.^[^ ^39^ ^]^ In embryonic stem cells, however, trimethylation levels of H3K9, H3K27, H3K36, and H4K20 were increased while mono‐methylation levels of these sites were reduced upon glutamine deprivation (loss of αKG anaplerosis) without affecting H3K4 histone methylation.^[^ ^40^ ^]^ Even in different kinds of tumor cells like 3T3, M229, Hela, and KP^sh^ (PDAC cell lines), the addition of cell‐permeable αKG or deletion of intracellular αKG exerted different demethylating effects on histone lysine sites.^[^ ^38^ ^]^ Our study confirmed that although αKG exhibited certain differences in demethylation capacity in three kinds of tumor cells (“hot” and “cold”), B2M was overexpressed in all the tumor cells derived from three distinct types of malignancies. After excluding possibilities of other histone methylation loci, we confirmed that H3K4me1 in the promoter region of B2M inhibited transcription and demethylation of H3K4me1 rather than H3K4me3 by adding αKG could enhance the transcription. Interestingly, further elevation of H3k4me1 pharmacologically with CPI455 or GSK‐J4 or by overexpressing KMT2D could not inhibit the transcription of B2M, while knockdown of KMT2D demethylated H3K4me1 and also achieved upregulation of B2M. H3K4 methylation and especially H3k4me1 in the distal enhancer region or near the transcription start sites have previously been associated with the activation of target genes.^[^ ^41^ ^]^ These results provide a fresh perspective on the relationship between histone methylation and transcription regulation.

It was previously reported that IDH1 and IDH2 mutation drives αKG loss and accumulation of its competitive inhibiting product: D‐2‐hydroxyglutarate (D‐2HG) and R‐2‐hydroxyglutarate (R‐2HG) in glioma.^[^ ^3^ ^]^ However, these efforts were focused on the metabolic and epigenetic disturbance of these oncometabolites in both tumor cells and T cells, while inhibition of these two derivatives could strengthen anti‐tumor immunity by restoring impairments of T cell differentiation and proliferation. Our findings instead demonstrated the distribution differences of αKG between tumors and normal tissues and further revealed the association of better prognosis and higher αKG contents by directly examining αKG concentration in clinical specimens of RCC. Moreover, supplementation of cell‐permeable αKG in tumor‐cell cultivation medium could significantly strengthen CD8^+^ T cell‐mediated killing effects on murine renal carcinoma cells without influencing tumor cell proliferation and apoptosis. Of note, treatment with αKG or transfection of B2M‐Overexpression plasmids both promoted expression of PD‐1 in CD8^+^ T cells and secretion of IFN‐γ and TNF‐α, the key indicators of anti‐tumor properties of CTLs.^[^ ^42^ ^]^ It still remains debatable whether B2M expression is associated with infiltration and effects of CD8^+^ T cells, CD4^+^ T cells or NK cells.^[^ ^43^ ^]^ Combining the results from the public database and our results in vitro and in vivo, we confirmed B2M overexpression of tumor cells could enhance the cytotoxic effects of CD8^+^ T cells and increase proportions of tumor‐infiltrating CD8^+^ T cells rather than CD4^+^ T cells.

The study has certain limitations, such as DNA methylation and histone acetylation modification are the main forms of epigenetic modification, and there is a need to further examine the role of histone acetylation. The number of clinical samples studied should be largely increased to investigate whether AKG can be a tumor prognosis/biomarker of RCC in the future.

In conclusion, we reveal an immunoregulatory circuit in which AKG inhibited RCC tumor growth by attenuating the enrichment of H3K4me1 in the promoter regions to upregulate B2M expression and enhance CD8^+^ T cell‐mediated anti‐tumor immunity.

## 4 Experimental Section

### Cells Lines

The human renal cancer cell lines HK‐2, 786‐O, A498, ACHN, CAKI‐1, and OS‐RC‐2, and murine prostate cell line RM‐1 were generously provided by Chen's lab, while the murine renal cancer cell line Renca and B16‐F10 were purchased from Procell Life Science & Technology.

### Human Samples

Resected human RCC tissues were obtained from patients at the Tongji Hospital (Wuhan). Ethical permission was granted by the Clinical Trial Ethics Committee of Tongji Hospital (Wuhan). All patients provided written informed consent to participate in the study (2019CR101).

### Animals

The study utilized male C57BL/6 and Balb/c mice, aged 6–8 weeks, procured from Cyagen Corporation and housed under pathogen‐free conditions at the Animal Facilities of Tongji Hospital experimental animal center. The Animal Care and Use Committee of Tongji Hospital approved all procedures involving the mice (TJH‐202208007). Subcutaneous injection of 1.0 × 10^6^ cells of B16F10 or Renca was administered to the lower right flank of male C57BL/6 (B16F10) or Balb/c (Renca) mice to induce tumor growth. Tumor sizes were monitored regularly using calipers, and at specific time points, tumors were collected, weighed, and evaluated for immune phenotypes via flow cytometry or immunofluorescence. To assess the efficacy of immune checkpoint antibodies, mice were grafted with B16F10 or Renca cells and treated with 200 µg control IgG [rat IgG2a; BE0089 (BioXcell)], anti‐CD279 (PD1) [BEIGENE, Beijing]. Antibodies were injected (i.p.) 3–5 times (every 3 days starting from the indicated date). To deplete CD8^+^ T cells, mice were treated with anti‐CD8^+^ antibody [2.43 (BE0061, BioXcell); controls were treated with 200 µg IgG [rat IgG2b (BE0090, BioXcell)]. Antibodies were injected (i.p.) every 3 days starting one day prior to tumor cell inoculation. To evaluate the percent survival of animals, mice with tumors exceeding 2000 mm^3^ were defined as “dead”.

### Western Blotting

To extract protein from the samples, triplicate wells were first lysed in chilled RIPA buffer that contained complete protease inhibitors and PhoSTOP phosphatase inhibitors (Cat: 20115ES60, Yeason, China). The protein concentration of the lysates was quantified using the BCA protein quantification kit (Cat: 20201ES76, Yeason, China). For SDS‐PAGE and western blotting analyses, the protein samples were loaded onto a gel after being suspended in Laemmli buffer and sonicated 15 times for 30 seconds with intermittent breaks. The samples were then boiled and used for western blot analysis following the addition of β‐mercaptoethanol and bromophenol blue. The membranes were blocked in EpiZyme fast‐blocking buffer and incubated overnight at 4°C with the primary antibody in blocking buffer containing 0.2% Tween‐20. Primary antibodies include anti‐B2M antibody (Cat: R23610, ZENBIO, China), anti‐β‐Actin antibody (Cat: AC004, ABCLONAL, China), anti‐H3 antibody (Cat: A2348, ABCLONAL, China) anti‐GDH1 antibody (Cat: A5176, ABCLONAL, China). Refer to Table S1 (Supporting Information) for more information. For the secondary antibody treatment, the membranes were either blocked in fast blocking buffer at room temperature for 10–15 minutes or incubated directly in the diluent‐blocking buffer containing 0.2% Tween‐20 and 0.01% SDS for 1 h at room temperature. The membranes were imaged using fluorescence on a Biorad Imager and processed using Adobe Photoshop CC 2018.

### RNA‐Seq, Chip‐Seq, and Bioinformatics Analysis

Sequencing service was provided by Bioyi Biotechnology Co., Ltd. Wuhan, China. Public RNA‐seq data were from Gene Expression Omnibus (GEO) under accession number GSE167514 and GSE121580. Gsea analysis was conducted using public tool EasyGEO (https://tau.cmmt.ubc.ca/eVITTA/).^[^ ^44^ ^]^ Schematic diagrams were drawn by Figdraw (https://www.figdraw.com/static/index.html).

### Statistics and Reproducibility

In vitro experiments were repeated at least three times and shown with representative data unless otherwise specified. All experiments in vivo were performed with a minimum of three biological replicates, yielding similar results in each experiment. A two‐tailed unpaired Student's *t*‐test was used to determine statistical significance between two groups, while one‐way ANOVA was used for more than two groups with a single experimental parameter. Two‐way ANOVA was used to assess statistical significance between groups with two experimental parameters. Survival curves were tested using a log‐rank (Mantel–Cox) test. Unless otherwise stated, error bars indicate the standard error of the mean (s.e.m). Results with *p*‐values < 0.05 were considered significant. The allocation of mice to control or experimental conditions was random.

### Data and Materials Availability

Mice RNA‐sequencing and Chip‐seq datasets were deposited to the Genome Sequence Archive (GSA) website (https://ngdc.cncb.ac.cn/gsub/) affiliated with the National Genomics Data Center (https://ngdc.cncb.ac.cn/) with identifier CRA011350 and CRA011351. All other data are available in the main manuscript or Supporting Information.

## Conflict of Interest

The authors declare no conflict of interest.

## Author Contributions

L.L., X.Z., and Z.C. contributed equally to this work. P.X.L., Z.H.W., X.Z., L.L., Z.C., G.H.W., and Z.Q.H. conceived and designed the study. L.L. and Z.C. performed the experiments in vivo and in vitro, analyzed the results, wrote the first draft of the manuscript, and completed the revision. L.L., Y.G., Y.N.W., Z.Z.X., and S.M. collected clinical samples and conducted followed‐up. Q.Z., J.B.Z., and J.H.T. assisted with data analysis. J.L., B.J.W., X.Z., Y.B., and H.D. assisted with language and figure quality improvement. G.H.W. and W.G. helped with revision and graphical arrangement.

## Supporting information

Supporting Information

## Data Availability

The data that support the findings of this study are available from the corresponding author upon reasonable request.
