# Supplementary material for: Targeting Alpha‐Ketoglutarate Disruption Overcomes Immunoevasion and Improves PD‐1 Blockade Immunotherapy in Renal Cell Carcinoma
Source: Adv Sci (Weinh). 2023 Aug 1;10(27):2301975. doi: 10.1002/advs.202301975 (PMC10520657; doi:10.1002/advs.202301975)
Supplement: Supplementary file 1 — Supporting Information [file ADVS-10-2301975-s001.pdf]

ADVANCED SCIENCE
Open Access

Supporting Information

for *Adv. Sci.*, DOI 10.1002/advs.202301975

Targeting Alpha-Ketoglutarate Disruption Overcomes Immunoevasion and Improves PD-1 Blockade Immunotherapy in Renal Cell Carcinoma

Le Li, Xing Zeng, Zheng Chao, Jing Luo, Wei Guan, Qiang Zhang, Yue Ge, Yanan Wang, Zezhong Xiong, Sheng Ma, Qiang Zhou, Junbiao Zhang, Jihua Tian, David Horne, Bertram Yuh, Zhiquan Hu, Gong-Hong Wei, Baojun Wang, Xu Zhang, Peixiang Lan and Zhihua Wang**

Targeting Alpha-Ketoglutarate Disruption Overcomes Immunoevasion and Improves PD-1 Blockade Immunotherapy in Renal Cell Carcinoma

Le Li[#12], Xing Zeng[#1], Zheng Chao[#12], Jing Luo[3], Qiang Zhang[4], Yue Ge[1], Yanan Wang[1], Zezhong Xiong[1], Sheng Ma[1], Qiang Zhou[1], Junbiao Zhang[1], Jihua Tian[1], David Horne[5], Bertram Yuh[5], Zhiquan Hu[1], Gonghong Wei[6], Baojun Wang[7], Xu Zhang[7], Peixiang Lan[2], Zhihua Wang[1]

Supplementary methods

LC-MS analysis

The endogenous α-ketoglutaric acid was determined by Wuhan Greensword Creation Technology Co. Ltd., (Wuhan, China) (http://www.greenswordcreation.com) with UPLC-MS/MS. To quantify accurately, [$^{13}C_5$] α-ketoglutaric acid was used as an internal standard and α-ketoglutaric acid and [$^{13}C_5$] α-ketoglutaric acid were detected in negative mode with triple quadrupole mass spectrometry (Thermo Scientific TSQ Quantiva). The transition was 145.1>101.0, 145.1>57.1 for α-ketoglutaric acid; 150.1>105.0, 150.1>60.1 for [$^{13}C_5$] α-ketoglutaric acid with sheath gas flow, 30 Arb; auxiliary gas flow, 10 Arb; sweep gas flow, 8 Arb; collision gas, 1.5 mTorr; ion transfer tube temperature, 350 °C; vaporizer temperature, 300 °C.

Cell Culture and Chemical Agents

To ensure the authenticity and quality of the cell lines, we subjected them to testing for Mycoplasma contamination and interspecies cross-contamination and authenticated them by isoenzyme and short-tandem repeat analyses in the Cell

Resource Center of Wuhan University prior to the study. We cultured 786-O, RM-1, and B16-F10 cells with RPMI-1640 (Gibco), supplemented with 2 mM Glutamax, 100 IU/mL Penicillin, 100 mg/mL Streptomycin, and 5% heat-inactivated fetal calf serum (HI-FCS). A498, ACHN, and CAKI-1 cells were cultured with DMEM (Gibco), supplemented with 2 mM Glutamax, 100 IU/mL Penicillin, 100 mg/mL Streptomycin and 5% heat-inactivated fetal calf serum (HI-FCS). A498, ACHN and CAKI-1 cells were cultured with DMEM (Gibco), supplemented with 2 mM Glutamax, 100 IU/mL Penicillin, 100 mg/mL Streptomycin and 10% HI-FCS. OS-RC-2 and HK-2 cells were cultured in DMEM/F12 (Gibco) with 15 mM HEPES, supplemented with 2 mM Glutamax (Invitrogen), 100 IU/mL Penicillin, 100 mg/mL Streptomycin, 4 mg/mL Hydrocortisone (Sigma-Aldrich), 5 ng/mL EGF (Invitrogen), 5 mL of Insulin-Transferrin-Selenium solution (Life Technologies) and 10% HI-FCS. Renca cells were cultured in a specific culture medium provided by Procell Life Science&Technology (CM-0568). All the mentioned cells were maintained at 5% CO2 at 37 °C.

Mouse lymphocytes were isolated from the spleen and lymph nodes, and CD8+ T cells were separated using magnetic sorting with anti-APC magnet beads (Miltenyi Biotec) and anti-APC CD8 antibody (Biolegend). The isolated CD8+ T cells were then re-suspended (106 cells/ml) and activated with anti-CD3 and anti-CD28 mAbs for 48 h. Intratumor CD45+ T cells and tumor cells from mice were isolated as follows: mononuclear cells from the whole tumor were first enriched by density gradient centrifugation using Percoll (Biosharp). Then, red blood cells were lysed in

Cell Lysis Solution (Biosharp). Intratumor CD45+ T cells and tumor cells were collected using magnetic sorting with anti-PE magnet beads (Miltenyi Biotec) and anti-PE CD45 antibody (Biolegend). More information of materials seen in **Table S1**.

Flow cytometry analysis

In order to analyze the mouse tissue samples by flow cytometry, the tumors were cut into small pieces in DMEM and digested with 2 mg collagenase (Sigma, USA) for one hour at 37°C. Using a 70mm nylon strainer, cells were resuspended in red blood cell lysis buffer (Biosharp) for 3 min at room temperature. Afterward, cells were suspended in PBS with 2% BSA and stained with the corresponding antibodies.

Quantitative PCR with reverse transcription

For RNA extraction, a Fastagen fast RNA extraction kit was used, and 1000 ng of RNA was used for complementary DNA synthesis with a cDNA synthesis kit from Takara. The cDNA was then diluted 1:10 for quantitative PCR (qPCR). The cDNAs were mixed with gene-specific primers listed in Supplementary Tables and SYBR green PCR Master Mix from Vazyme, and qRT-PCR was performed on an Applied Biosystems 8100HT Fast Real-Time PCR system. The values were normalized to the expression of the housekeeping gene β-actin. For qPCR of tumor cells derived from murine models in vivo, we separated the tumor mass, grinded physically, filtered with cell filter and then performed the gradient centrifugation using a density of 38% Percoll. After lysing red blood cells, we collected the mixture of immune cells and tumor cells, separated tumor cells (CD45 negative) after magnetic beads sorting and

undertook qPCR analysis of these tumor cells.

Primer sequence: qRT-PCR primers seen in **Table S2**; shRNA primers seen in **Table S3**.

Immunohistochemistry

To prepare the tissue arrays for analysis, we first deparaffinized them by baking them at 60 °C for 30 minutes, followed by a series of washes with Citrus Clearing Solvent, 100% ethanol, 95% ethanol, and 70% ethanol. To retrieve antigens, we subjected the slides to high temperatures in a pressure cooker with pH 6.0 citrate (Vector Laboratories) for 30 seconds at 125 °C, followed by 10 seconds at 90 °C. Slides were permeabilized with 0.1% TBS-T for 5 minutes, blocked with Bloxall peroxidase block for 10 min and horse serum for 20 minutes. Next, we incubated the arrays overnight with the primary antibody and subsequently incubated them with the secondary antibody for 30 minutes at room temperature. We then applied the Vectastain Elite ABC reagent for 30 minutes and a 1:1 mixture of ImPACT DAB EqV reagent 1 (Chromogen) and reagent 2 (Diluent). After washing the slides with water three times, we applied the bluing reagent once, washed the slides once with water, and dehydrated the tissue by washing them five times with 70% ethanol, followed by ten times with 95% ethanol and twice for 10 minutes with 100% ethanol. Finally, we mounted the slides with Cytoseal 60 (Biosharp) and captured images using a BioTek Cytation 5.

Immunofluorescence analysis

Cells were seeded onto fibronectin-coated glass chamber slides (Biosharp) and then fixed in 4% paraformaldehyde at room temperature for 15 minutes. Following fixation, the samples were blocked with Gelatin Block (10% normal donkey serum, 1% BSA, 0.5% Triton X-100 in PBS) at room temperature. Next, the samples were incubated with the primary antibody overnight at 4 °C or for 1 hour at room temperature, followed by washing with PBS and exposure to the secondary antibody for 1 hour at room temperature. Cells were then stained with DAPI (Servicebio) before imaging. The antibodies and their corresponding dilutions were used in accordance with the manufacturer's instructions.

CCK-8 assay

Renca cells after corresponding treatment in different groups were respectively seeded in 96-well culture plates (1×10^4/well) and incubated overnight at 37°C in a 5% CO2 incubator. The cells inoculated the day before were washed twice with PBS and then added with 100μl 1640 medium. Discard the old solution at 0, 24, 48 and 96 h after liquid exchange. Add 90μl medium and 10ul CCK-8 to each well of the 96-well test plate and incubate at 37°C for 2 h. A microplate reader (BioTek Instruments, Inc., Winooski, VT, USA) was used to measure OD values at 450nm for each experimental well and to detect changes in cell proliferation ability in each group.

Cell apoptosis assay

Apoptosis was analyzed using a flow cytometry assay (Becton Dickinson). For co-cultivation experiments, we first collected lymphocytes from Balb/c spleens, and then collected $CD8^+$ T cells purified by positive selection with magnetic beads from

lymphocytes isolated from Balb/c mouse spleen and stimulated them with anti-CD3, anti-CD28 antibody and β-mercaptoethanol for 48 hours.

Cell Transfection

Plasmids V101-3flag and PCDH-H1 were gifts from the Laboratory of Biliary and Pancreatic Disease in Tongji hospital. Neofect was used to transfect the plasmids. Lentivirus-based short hairpin RNAs (shRNAs) were purchased from Sigma (USA). For RNA interference, gene-specific shRNA in combination with PSPAX and PMD2G were transfected into 293T cells. Twenty-four hours post transfection, the culture medium was replaced with fresh culture medium with 10% FBS. After 24 h, the medium without 293T cells was harvested, filtered and mixed with the medium containing cultured tumor cells. 3 days later, puromycin (10 μg mL^{-1}) was used to select positive renal cancer cells infected with shRNAs.

Chromatin immunoprecipitation and Chip-seq

Following 1% formaldehyde cross-linking, chromatin was isolated and sonicated using a Diagenode Bioruptor Pico sonicator to generate fragments with a size range of 300-700 base pairs. For immunoprecipitation, 250 μl of the fragmented chromatin was incubated overnight at 4°C with 5 μg of anti-H3K4me1 antibody (Sigma-Aldrich 17-10489) or normal rabbit IgG antibody (12-370) as a control, bound to protein A/G coated magnetic beads with rotation. The DNA was then reverse-crosslinked and eluted from the protein-DNA complex using an elution buffer (1% SDS, 100mM NaHCO3) while shaking at 65 °C for 1 hour. The purified DNA was extracted using

the Maxtract tube (QIAGEN) with a phenol-chloroform extraction method, followed by overnight settling of ethanol. The immunoprecipitated DNA was further processed for sequencing using Illumina technology with modified TruSeq adapters and NEBNext ChIP-Seq Library Prep Master Mix Set.

Supplementary figures, tables and figure legends

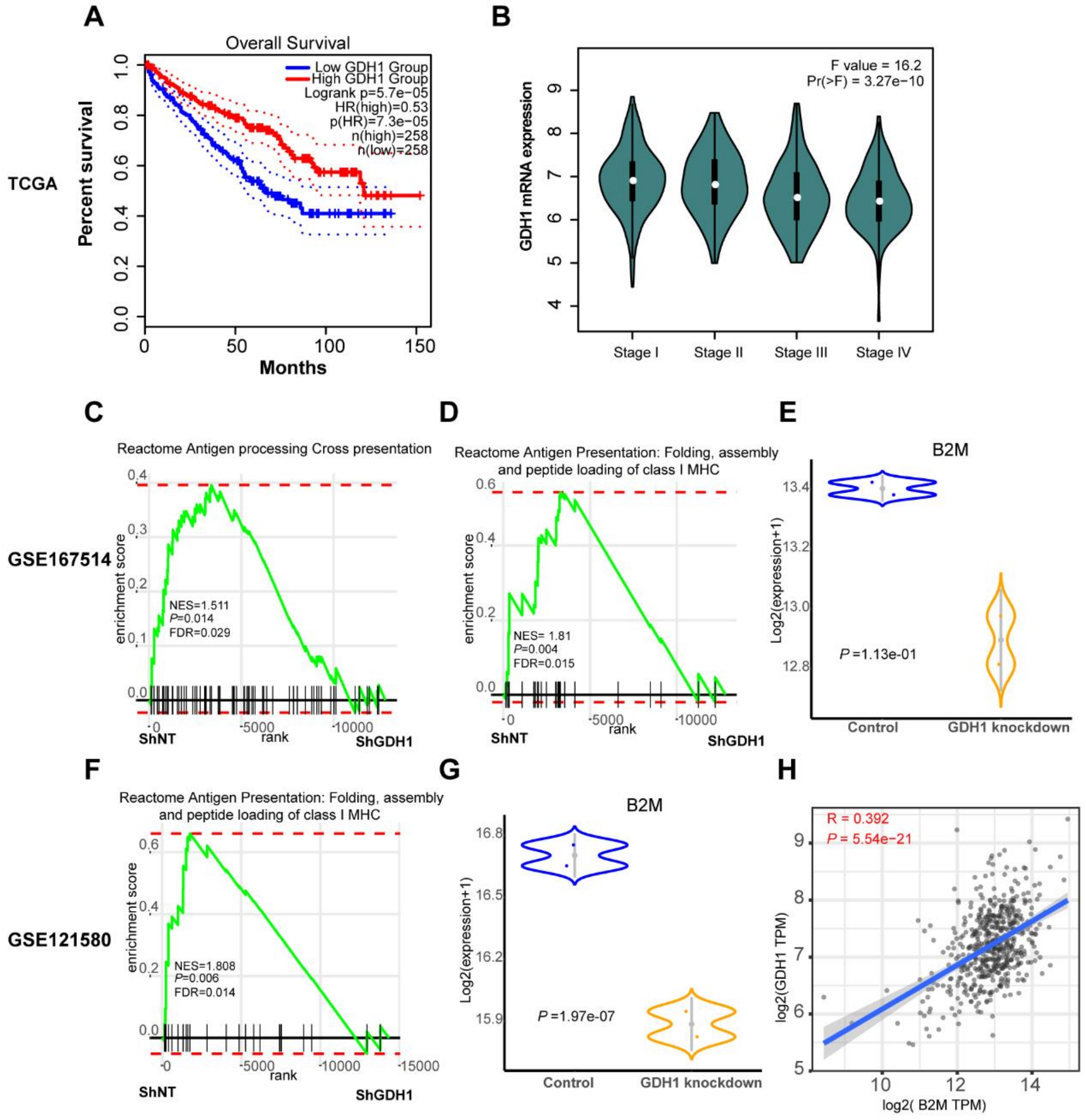

Figure S1. GDH1 is positively correlated with the prognosis and B2M expression. (A) Overall survival and **(B)** tumor stages of TCGA-KIRC patients with high or low GDH1 expression. **(C-E)** Gsea results and B2M expression in the control group and ShGDH1 group in human 786O cells

(GSE167514) and **(F-G)** in human U87 cells (GSE121580). Data was analyzed using EasyGEO database. **(H)** Correlation of B2M expression with GDH1 expression in KIRC obtained from TIMER (purity-corrected Spearman test).

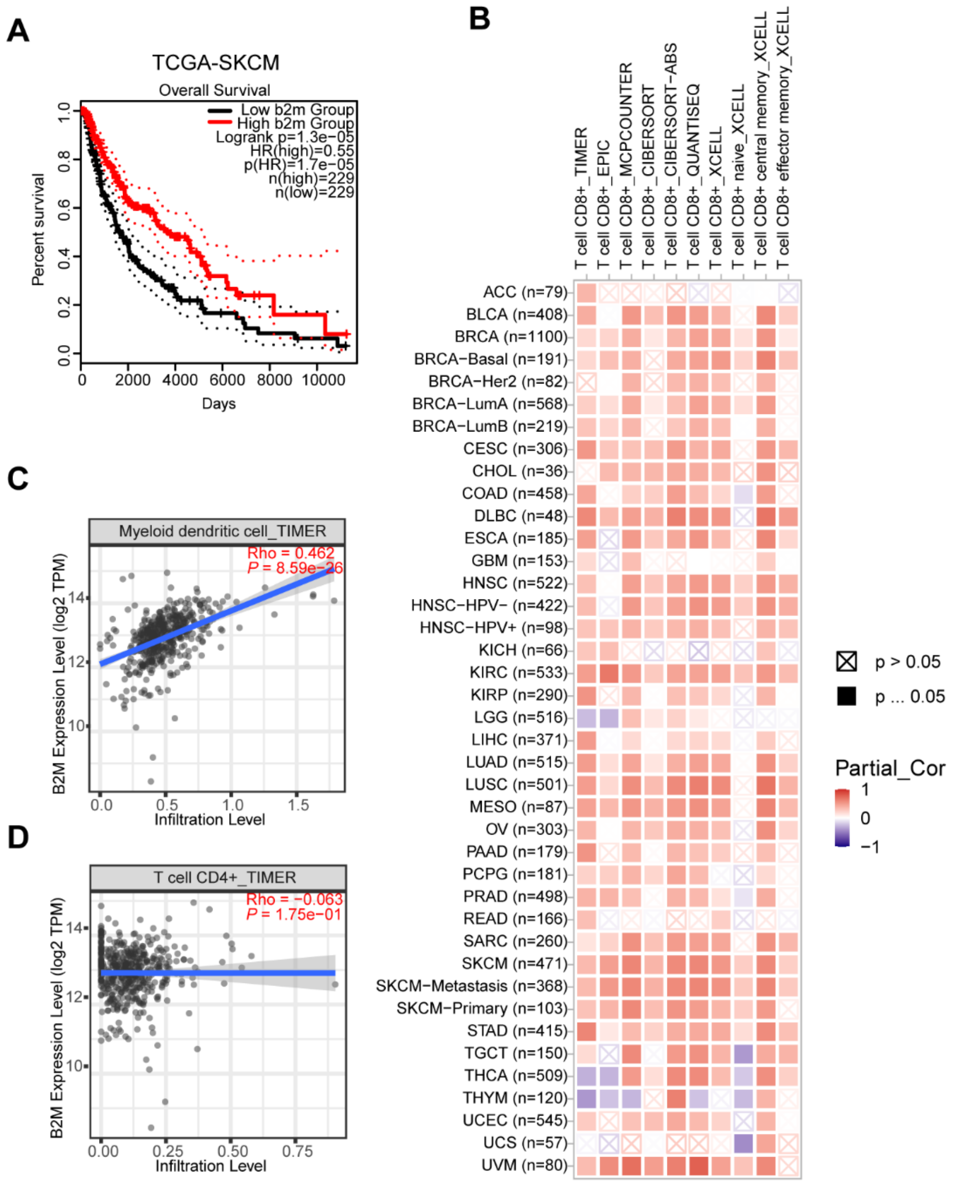

Figure S2. High expression of *B2M* predicts better survival and correlates with CD8^{+} T cell infiltration. (A) Overall survival of TCGA-SKCM patients with high or low B2M expression. **(B)** Correlation of B2M expression and tumor-infiltrating CD8^{+} T cell proportions in multiple tumors of TCGA. **(C-D)** Correlation of B2M expression and tumor-infiltrating DCs and CD4^{+} T cell proportions in TCGA-KIRC. Immune data were analyzed using Timer database.

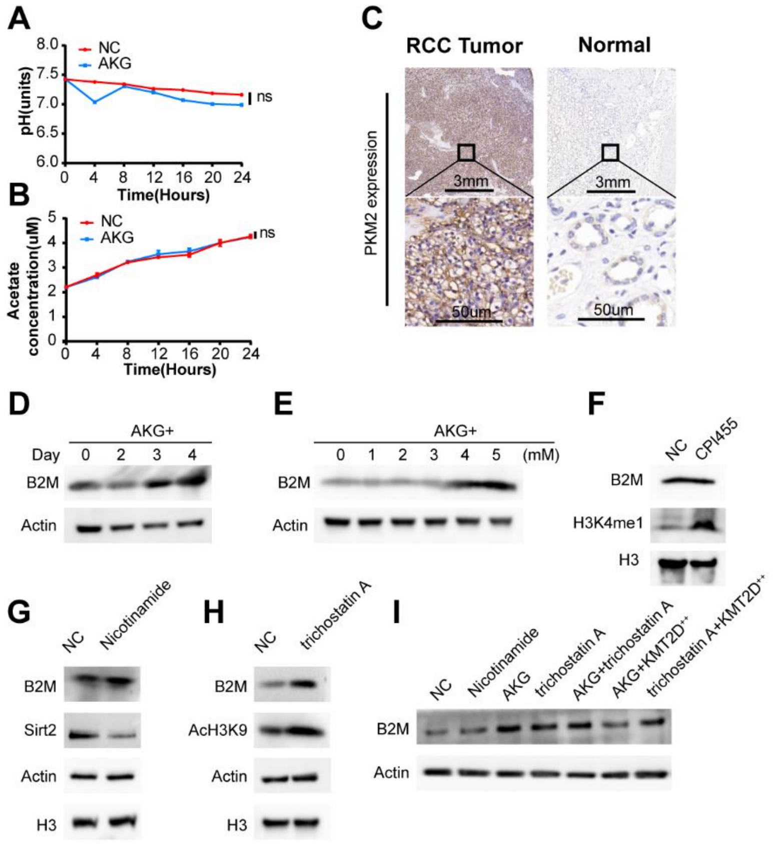

Figure S3. AKG promoted B2M expression in Renca cells. (A) pH values and **(B)** acetate levels of cell media after αKG/PBS (NC) supplementation within 24 hours. **(C)** PKM2 expression in clinical RCC tumor sanples and adjacent normal tissues using IHC analysis. **(D)** Expression of B2M in Renca cells after αKG supplementation at different time. **(E)** Expression of B2M in Renca cells after αKG supplementation with different doses. **(F)** Expression of B2M and H3K4me1 in Renca cells after CPI455 supplementation. **(G)** Expression of B2M and Sirt2 in Renca cells after αKG Nicotinamide supplementation. **(H)** Expression of B2M and AcH3K9 in Renca cells after trichostatin A supplementation. **(I)** Expression of B2M in Renca cells after αKG, Nicotinamide and/or trichostatin A supplementation with/without KMT2D overexpression ($KMT2D^{++}$).

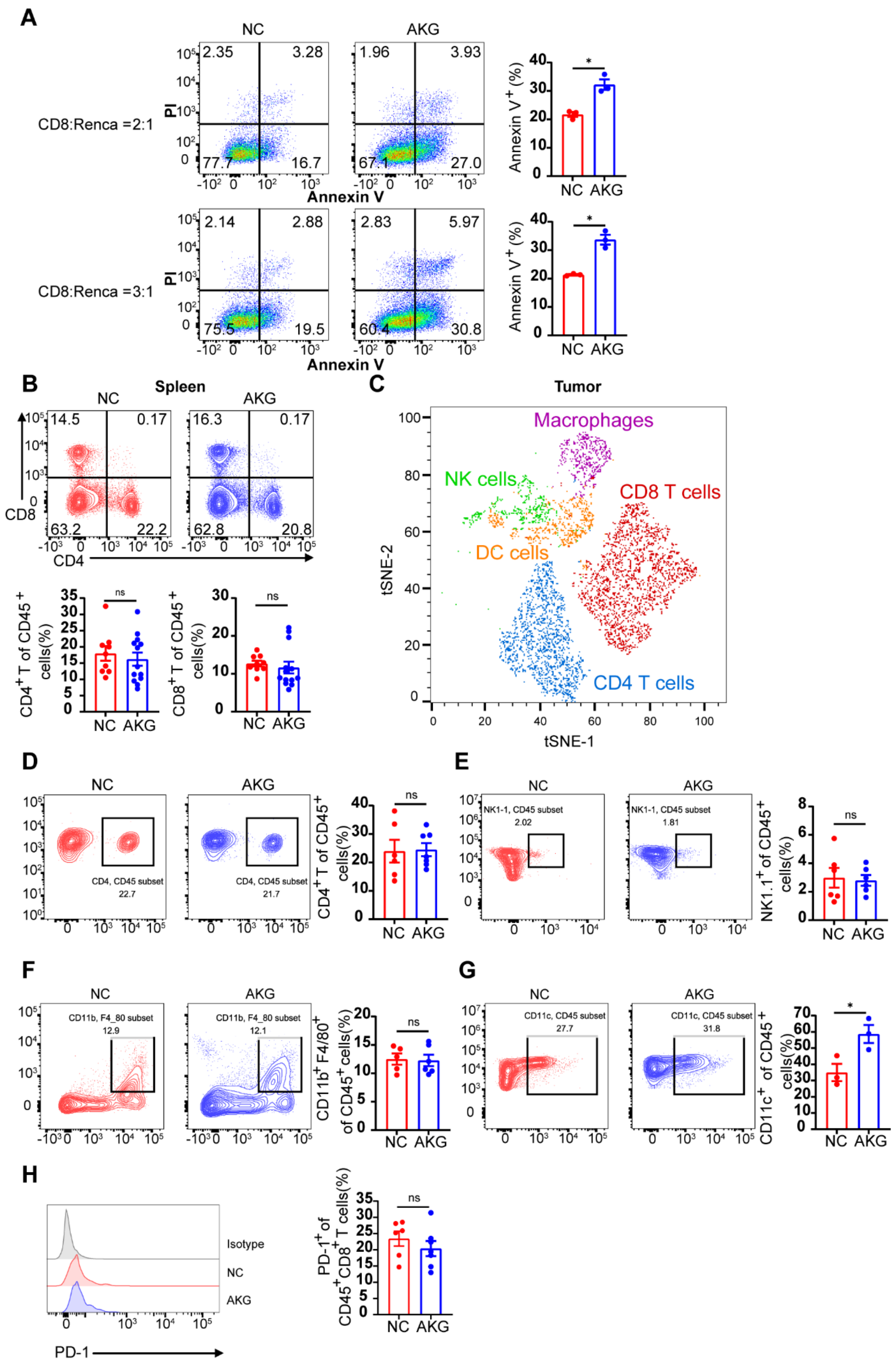

Figure S4. AKG supplementation augmented CTL cytotoxicity to tumor cells and improved tumoral infiltration of DCs. (A) Representative flow plots and quantification of Renca cell proportions in the αKG group treated with 5mM αKG and control group after co-cultivation with CD8+

T cells at a ratio of 1:2 and 1:3 for 24 hours. **(B)** Representative flow plots and quantification of $CD4^+$ and $CD8^+$ T cells in spleens of mice subcutaneously implanted with Renca tumors of the αKG group (n=13) and control group(n=9). **(C)** tSNE clustering of murine intratumoral immune cell subsets. **(D)** Representative flow plots and quantification of tumor-infiltrating $CD4^+$ T cells (n=6 for the control group and n=7 for αKG group), **(E)** NK cells (n=6), **(F)** macrophages (n=5 for the control group and n=6 for αKG group) and **(G)** DCs (n=3) in Renca tumors subcutaneously implanted. **(H)** Representative flow plots and quantification of PD-1 expression on the intra-tumoral $CD4^+$ T cells of the control group(n=6) and αKG group (n=7) from Renca tumor-bearing mice.

Table S1. Materials information

REAGENT or RESOURCE	SOURCE	IDENTIFIER
[13C5]-α-Ketogultaric Acid	ISOREAG	IR-33576
α-Ketogultaric Acid	ISOREAG	IR-47108
PHENOL	ALADDIN	P128316
CHLOROFORM-D	ALADDIN	C434287
Percoll	BIOSHARP	BS909
Red Blood Cell Lysis Buffer	SERVICEBIO	G2015
Anti-APC MicroBeads	MILTENYI BIOTEC	130-090-855
Anti-PE MicroBeads	MILTENYI BIOTEC	130-048-801
APC anti-mouse CD45	BIOLEGAND	103112
RNAfast 200	FASTAGEN	220011
REVERSE TRANSCRIPTASE	YEASEN	11111ES92
qPCR SYBR Green Master Mix	YEASEN	11200ES03
Phosphate Buffered Saline	SERVICEBIO	G4202
beta 2 Microglobulin Rabbit mAb	ZENBIO	R23610
beta 2 Microglobulin Mouse mAb	ZENBIO	220909
β-Actin Mouse mAb	ABCLONAL	AC004
Goat Anti-Rabbit IgG H&L HRP	ZENBIO	511203
Protein Free Rapid Blocking Buffer	EPIZYME	PS108P
RIPA Lysis Buffer	SERVICEBIO	G2002
PSMF	THERMO	36978
ProteinSample Loading Buffer	EPIZYME	LT101
180kDa Prestained Protein Marker	VAZYME	MP102
Omni-ECL Femto Light Chemiluminescence Kit	EPIZYME	SQ201
Tris-base Saline	SERVICEBIO	G0015
Twee 20 Surfact-Amps	THERMO	85113
PVDF Transfer Membrane	MERCK	IPVH00010
FuturePAGE™ 8% 12 Wells	ACE	ET12008
Skim Milk Powder	BIOSHARP	BS102
Paraformaldehyde Fixative	SERVICEBIO	G1101
Triton-X	BIOSHARP	BS084
Normal Goat Serum	CELL SIGNALING TECHNOLOGY	5425

Anti-5-hydroxymethylcytosine	ABCAM	AB214728
Anti-rabbit IgG (H+L), F(ab')2 Fragment	CELL SIGNALING TECHNOLOGY	4413
Anti-mouse IgG (H+L), F(ab')2 Fragment	CELL SIGNALING TECHNOLOGY	4408
Hoechst 33258 Staining Solution	SERVICEBIO	G1011
BioDewax and Clear Solution	SERVICEBIO	G1128
Ethanol absolute	SINOPHARM	10009218
Citric Acid Antigen-Retrieval Solution	SERVICEBIO	G1202
Hydrogen peroxide	SINOPHARM	10011208
Beta-2-Microglobulin Polyclonal antibody	PROTEINTECH	13511-1-AP
Beta Actin Polyclonal antibody	PROTEINTECH	20536-1-AP
PD-1/CD279 Polyclonal antibody	PROTEINTECH	18106-1-AP
CD8 Monoclonal antibody	PROTEINTECH	66868-1-Ig
DAB Chromogenic Kit	SERVICEBIO	G1212-200T
Haematoxylin Solution	SERVICEBIO	G1004
Hematoxylin Bluing Solution	SERVICEBIO	G1040
Neutral Tree Gum	SERVICEBIO	WG10004160
D-2HG	CAYMAN CHEMICALS	11605
Mono-Methyl-Histone H3 (Lys4) (D1A9) Rabbit mAb	CELL SIGNALING TECHNOLOGY	5326
Tri-Methyl-Histone H3 (Lys4) (C42D8) Rabbit mAb	CELL SIGNALING TECHNOLOGY	9751
Di-Methyl-Histone H3 (Lys9) (D85B4) Rabbit mAb	CELL SIGNALING TECHNOLOGY	4658
Tri-Methyl-Histone H3 (Lys9) (D4W1U) Rabbit mAb	CELL SIGNALING TECHNOLOGY	13969
Tri-Methyl-Histone H3 (Lys27) (C36B11) Rabbit mAb	CELL SIGNALING TECHNOLOGY	9733
Histone H3 (3H1) Rabbit mAb	CELL SIGNALING TECHNOLOGY	9717
BIX01294	BEYOTIME	SD1123
UNC1999	MEDCHEMEXPRESS	HY-15646
CPI-455	TARGETMOI	T3552
JIB-04	MEDCHEMEXPRESS	HY-13953
GSK-J4	MEDCHEMEXPRESS	HY-15648B
AG120	BEYOTIME	SC6574
GSK126	BEYOTIME	SC0060
NV03	MEDCHEMEXPRESS	HY-125292
UNC0631	MEDCHEMEXPRESS	HY-13808
MRK-740	MEDCHEMEXPRESS	HY-114209
PFI-2	MEDCHEMEXPRESS	HY-18627

Anti-Mono-Methyl-Histone H3 (Lys4) Mouse mAb	PTM BIOLABS	PTM-5158
Formaldehyde aqueous solution	SINOPHARM	10010061
Glycine	SERVICEBIO	GC304019
Cell Counting Kit-8	BIOSHARP	BS360A
Annexin V-FITC/PI apoptosis kit	MULTISCIENCES	AP101
PE anti-mouse CD8	BIOLEGAND	553032
InVivoMab anti-mouse CD3 ε	BIOXCELL	BE0001-1
InVivoMab anti-mouse CD28	BIOXCELL	BE0015-5
2-Mercaptoethanol	MERCK	M3148
Ficoll	DAKEWE	7211011
BV510 anti-mouse CD4	BIOLEGAND	100449
APC-Cy7 anti-mouse CD8	BIOLEGAND	100713
BV605 anti-mouse PD1/CD279	BIOLEGAND	563059
FITC anti-mouse Granzyme B	BIOLEGAND	396404
PE anti-mouse IFN-γ	BIOLEGAND	505808
FITC anti-mouse TNF-α	BIOLEGAND	506304
InVivoMab anti-mouse CD8α	BIOXCELL	BE0061
anti-murine PD -1 antibody in vivo	BEIGENE	MATERIAL TRANSFER
anti-murine Ig-G antibody in vivo	BEIGENE	MATERIAL TRANSFER
BV65 anti-mouse NK1.1	BIOLEGAND	108736
PE-Cy7 anti-mouse CD11b	BIOLEGAND	101216
BV421 anti-mouse CD11c	BIOLEGAND	117343
PE anti-mouse F4/80	BIOLEGAND	123110
DMEM basic	GIBCO	8122122
RPMI Medium 1640 basic	GIBCO	8122012
Fetal Bovine Serum	GIBCO	10099
Trypsin-EDTA Solution	BIOSHARP	BL512A

Table S2. Primer information

Species	Gene	Forward (5’-3’)	Reverse (5’-3’)
Mouse	β-actin	GAGACCTTCAACACCCCAGC	ATGTCACGCACGATTTCCC
Mouse	B2M	TTCTGGTGCTTGTCTCACTGA	CAGTATGTTCGGCTTCCCATTC
Mouse	TAP-1	GGACTTGCCTTGTTCCGAGAG	GCTGCCACATAACTGATAGCGA
Mouse	TAP-2	CTGGCGGACATGGCTTTACTT	CTCCCACTTTTAGCAGTCCCC
Mouse	Nlrc5	GCTGAGAGCATCCGACTGAAC	AGGTACATCAAGCTCGAAGCA
Mouse	TAPBP	GGGTGGCCTGTCTAAGAAACC	ACTCAGACTTCTAGCCCACTTC
Mouse	H2-K^{d}	TCCTGCTGTTG GCGGCCGCCC	GATGAGAAGGAGAAACACAG
Mous	KMT	GTGGCTGTTCCACACCCAG	AGCTTGAGCTTCTCAGCATCG

e	2D		
Human	β-actin	TGACGTGGACATCCGCAAAG	CTGGAAGGTGGACAGCGAGG
Human	B2M	GAGGCTATCCAGCGTACTCCA	CGGCAGGCATACTCATCTTTT

Table S3. ShRNA information

shKMT2D-F	5′-GATCGTTCATCGAGTTGCGACATAACTCGAGTTATGTCGCAACTCGATGAACTTTTTG-3′
shKMT2D-R	5′-GGCCCAAAAAGTTCATCGAGTTGCGACATAACTCGAGTTATGTCGCAACTCGATGAAC-3′
shB2M-1-F	5′-GATC AGAGTTAAGCATGCCAGTATGCTCGAGCATACTGGCATGCTTAACTCTTTTTTG-3′
shB2M-1-R	5′-GGCCCAAAAAAGAGTTAAGCATGCCAGTATGCTCGAGCATACTGGCATGCTTAACTCT-3′
shB2M-2-F	5′- GATCGCCGAACATACTGAACTGCTACTCGAG TAGCAGTTCAGTATGTTCGGC TTTTTG-3′
shB2M-2-R	5′-GGCCCAAAAAGCCGAACATACTGAACTGCTACTCGAG TAGCAGTTCAGTATGTTCGGC-3′
